# Perceived Usefulness, Competency, and Associated Factors in Using District Health Information System Data Among District Health Managers in Tanzania: Cross-sectional Study

**DOI:** 10.2196/29469

**Published:** 2022-05-23

**Authors:** Daudi Simba, Felix Sukums, Claud Kumalija, Sarah Eden Asiimwe, Sai Kumar Pothepragada, Patrick Warui Githendu

**Affiliations:** 1 Department of Community Health Muhimbili University of Health and Allied Sciences Dar es Salaam United Republic of Tanzania; 2 Directorate of Information and Communication Technology Muhimbili University of Health and Allied Sciences Dar es Salaam United Republic of Tanzania; 3 Health Management Information System Unit Ministry of Health, Community Development, Gender, Elderly and Children Dodoma United Republic of Tanzania; 4 The Global Fund to Fight HIV, Tuberculosis and Malaria Geneva Switzerland

**Keywords:** DHIS2, Tanzania, health information system, health management information system, perception, competency, usefulness

## Abstract

**Background:**

Tanzania introduced District Health Information Software (version 2; DHIS2) in 2013 to support existing health management information systems and to improve data quality and use. However, to achieve these objectives, it is imperative to build human resource capabilities to address the challenges of new technologies, especially in resource-constrained countries.

**Objective:**

This study aimed to determine the perceived usefulness, competency, and associated factors in using DHIS2 data among district health managers (DHMs) in Tanzania.

**Methods:**

This descriptive cross-sectional study used a quantitative approach, which involved using a self-administered web-based questionnaire. This study was conducted between April and September 2019. We included all core and co-opted members of the council or district health management teams (DHMTs) from all 186 districts in the country. Frequency and bivariate analyses were conducted, and the differences among categories were measured by using a chi-square test. *P* values of <.05 were considered significant.

**Results:**

A total of 2667 (77.96%) of the expected 3421 DHMs responded, of which 2598 (97.41%) consented and completed the questionnaires. Overall, the DHMs were satisfied with DHIS2 (2074/2596, 79.83%) because of workload reduction (2123/2598, 81.72%), the ease of learning (1953/2598, 75.17%), and enhanced data use (2239/2598, 86.18%). Although only half of the managers had user accounts (1380/2598, 53.12%) and were trained on DHIS2 data analysis (1237/2598, 47.61%), most claimed to have average to advanced skills in data validation (1774/2598, 68.28%), data visualization (1563/2598, 60.16%), and DHIS2 data use (1321/2598, 50.85%). The biggest challenges facing DHMs included the use of a paper-based system as the primary data source (1890/2598, 72.75%) and slow internet speed (1552/2598, 59.74%). Core members were more confident in using DHIS2 compared with other members (*P*=.004), whereas program coordinators were found to receive more training on data analysis and use (*P*=.001) and were more confident in using DHIS2 data compared with other DHMT members (*P*=.001).

**Conclusions:**

This study showed that DHMs have appreciable competencies in using the DHIS2 and its data. However, their skill levels have not been commensurate with the duration of DHIS2 use. This study recommends improvements in the access to and use of DHIS2 data. More training on data use is required and should involve using cost-effective approaches to include both the core and noncore members of the DHMTs. Moreover, enhancing the culture and capacity of data use will ensure the better management and accountability of health system performance.

## Introduction

### Background

Health information systems (HISs) are 1 of the 6 building blocks of the health system [1] and are crucial in addressing health challenges and improving health service delivery in resource-limited countries [2]. However, many resource-limited countries face challenges in improving their HISs using information and communication technology (ICT) [3]. The advent of a free and open-source web-based District Health Information Software (version 2; DHIS2) signaled a new era by providing a solution for addressing one of the major challenges in HISs [4], that is, the availability of quality and timely data for planning and evaluating health sector performance [5].

DHIS2 is a tool for the collection, validation, analysis, and presentation of aggregate data, tailored to facilitate integrated health information management activities and inform decision-making [3,6]. The software is based at the district level and derives primary data from paper-based monthly reports of health facilities. As a web-based system in a centralized server, DHIS2 presents a more stable solution compared with the previous HIS solutions [7]. With DHIS2, anyone with a computing device connected to the internet, a system username, and a password can view data anywhere as soon as it is entered [8].

The DHIS2 is a simplified generic tool based on an open metadata model and a flexible user interface that does not require a preconfigured database application [9]. Users can customize the software package according to their information needs without the need to have advanced programming skills or learn complex programming language [8-10]. The ultimate aim of DHIS2 is to establish a centralized database that connects service delivery with other health systems [11].

DHIS2 has several modules and features that make it superior to the previous HIS software used in sub-Saharan African countries [9]. Its features include customizable data entry forms with indicators; data visualizers, such as graphs, web-based pivot tables, and dashboards; an integrated geographic information system module; import and export metadata; customizable data quality checks; user access control; and messaging and mobile solutions. Other modules include a patient tracker and mobile app and are interoperable with other systems [12].

### The DHIS2 in Tanzania

Over the past decade, the Tanzanian health sector has seen an increasing focus on strengthening its national HIS. This followed a wind of change that swept across other African countries south of the Sahara because of accelerated disease control initiatives, specifically HIV, tuberculosis, and malaria, which created additional demand by partners for timely measurement of results [11].

Since then, several reviews have been conducted to identify bottlenecks and address them to improve HISs. The review conducted before the introduction of the DHIS2 showed that reporting systems were weak, both in terms of completeness and timeliness [13].

Consequently, the Ministry of Health, in collaboration with implementing and development partners who support various health service activities, including the DHIS2 implementation, created the Monitoring and Evaluation Strengthening Initiative in 2010, whose vision was to have a comprehensive platform for health information, evidence-based decision-making, and accountability for results [14]. The key strategic objective was the introduction of the DHIS2. Tanzania adopted the DHIS2 as a national health management information system (HMIS) platform in 2010, and by December 2013, all districts in the country were using the system. The system was customized to capture and process all routine aggregate data collected monthly from all health facilities, including public and nonpublic facilities [15,16].

ICT has an impact when people have the necessary competencies to develop and use it; otherwise, the system becomes a waste of resources [17]. Although ICT has been introduced to help manage resources and increase efficiency, many resource-limited countries face a severe shortage of appropriate human resources, both in terms of quantity (numbers) and quality (skills) [6,15,18]. The implementation of the DHIS2 in Tanzania, as in other resource-constrained countries that have adopted the system, is likely to suffer from poor and unevenly developed infrastructure, inadequate competencies among the workforce to support the system, and the use of information for decision-making [11]. To maximize return on investment, implementers of the DHIS2 need to address the major challenges, including skill deficiencies in the workforce [18]. Other factors affecting the successful implementation of an electronic HISs include positive attitudes toward the HISs, confidence in using computers and the HISs, and fast and reliable internet connectivity. These factors need to be regularly monitored, and challenges affecting the effective use of an electronic HISs must be addressed to promote a data culture for evidence-informed decision-making [19]. However, there is limited information on district health managers’ (DHMs) perceptions and competencies in using the DHIS2 and its data for informed decision-making in Tanzania.

## Methods

### Aim of This Study

This study aimed to determine the perceived usefulness of DHIS2 and its competency in using generated data among DHMs, who are the primary target beneficiaries of the system.

### Study Design

This is a descriptive cross-sectional analytic study that used a quantitative approach to gather information on the perceived usefulness of the DHIS2, the competencies of DHMs, and factors associated with the use of DHIS2 data to improve health services. Factors associated with DHIS2 use, according to the Performance of Routine Information System Management framework, were categorized as technical, organizational, and behavioral issues [20]. Respondents were not only given an opportunity to select from predefined factors but also given the freedom to specify other issues affecting the use of the DHIS2 and its data.

### Study Setting

Tanzania is an East African country made up of the mainland and islands of Zanzibar and is the largest country in the East African Community. It spreads over 947,300 km^2^ of land, is multiethnic, and has wide cultural diversity. At the time of this study, Tanzania was a low-income country, with a gross domestic product per capita of 957 and a growth rate of 6.8%. The Tanzania Mainland alone is estimated to have a population of 55 million, distributed in 26 regions and 186 districts. The country has >8000 health facilities, offering a wide range of health care services [21].

The health system in Tanzania is well organized in a hierarchical structure from the community to the national level. It is a decentralized system, whereby local government authorities are responsible for the planning and management of primary health service delivery. At the primary level, there are district hospitals, health centers, and dispensaries. The DHMs are responsible for district health planning, resource allocation, implementation of health interventions, and supervision of health service provision in primary health care settings. Thus, it is paramount to assess the perception and competencies of DHMs regarding the use of the DHIS2 and its data in accomplishing their core functions.

We defined the use of DHIS2 to include the entire process from data entry, navigation through the DHIS2 application, data validation, data analysis, and production of visualizations. We defined data use as the reported use of DHIS2 data for the planning and implementation of activities, for example, for planning, staff deployment, allocation of funds, procurement of medicine and supplies, and production of league tables.

### Study Population

All core and co-opted members of the district health management teams (DHMTs) were included. Core members were recruited based on their professional cadre. These included the district medical officer, who is a medical doctor, and the district health officer, who is an environmental health scientist by profession. Co-opted members are program coordinators who are invited to the DHMT from time to time when issues pertaining to their programs are discussed. These include the reproductive and child health, HIV, tuberculosis, and malaria programs.

### Sampling Strategy and Sample Size

Data were collected to represent the entire country; hence, 186 districts in all 26 regions in the country were included. In each district, both core and co-opted members of the DHMTs (approximately 15) were interviewed.

At the beginning of this study, the researchers (DS, FS, and CK) collected the names, email addresses, and phone numbers of all DHMs from among the regional HMIS focal persons. Data managers linked DHMs to the web-based questionnaire through their email addresses, and phone numbers were used to follow up on nonresponders and to communicate data discrepancies. The email addresses were collected on a separate sheet and used only by the data managers for follow-up during data collection. No identifiable personal information was included in this questionnaire.

### Data Collection Approaches

This study was conducted between April and September 2019. We developed a questionnaire containing different aspects of the DHIS2, data management and use. The questionnaire had several sections, including sociodemographic information, respondents’ self-rated skill levels on basic computer applications and the DHIS2, and skills on data use for various purposes. The questionnaire also had a section that assessed perceived usefulness, ease of use, and user satisfaction with the DHIS2 [22]. The perception statements were rated on a 5-point Likert scale, ranging from 1 (strongly disagree) to 5 (strongly agree), covering managers’ experience and perception of the application of the DHIS2 in their work, its perceived benefits, and their willingness and ability to use the DHIS2 and data from the system for various decision-making processes. The availability of ICT support from the councils as well as challenges hindering the implementation and use of DHIS2 were also included in the questionnaire.

The questionnaires, translated into Kiswahili, were formatted using Google Forms to ease the filling and submission by respondents via an internet-enabled device with an internet browser. Furthermore, the questionnaires had mandatory quality checks to ensure that all relevant fields had been filled in before submission of the data into a database.

A total of 26 field staff members, 1 from each of the 26 regions, were recruited to facilitate data collection. The field staff comprised regional HMIS focal persons, who are government officials assigned with the responsibility to oversee HMIS activities in their respective regions. The regional HMIS focal persons received a half-day orientation on the study objectives and data collection procedures during the 2019 annual regional HMIS focal persons meeting. They were also tasked to provide the names and addresses of members of all DHMTs in their respective regions. Data managers later used these to send questionnaires to the respondents, who had to provide consent, fill in the questionnaire, and submit their responses on the web to a server located at the Muhimbili University of Health and Allied Sciences (MUHAS) offices. Field staff made follow-up visits to the respective districts to ensure the completion of the questionnaires.

Data managers and researchers (DS and FS) scrutinized the submitted data daily for duplicate submissions, incomplete questionnaires, and discrepancies. When duplicate forms were dropped, incomplete questionnaires and discrepancies were communicated to the respondents before making the necessary corrections in the database. In addition, the types of errors observed were communicated to the field staff, who used this information to guide respondents on proper filling in of the questionnaires during on-site follow-up visits. This helped to avoid the recurrence of similar mistakes.

The questionnaire was pretested with staff at Muhimbili National Hospital and MUHAS in Dar es Salaam to ascertain the ease of filling in the questionnaires and to ensure that the questions were clearly understood. The findings from the pretest were used to improve on the tools before distributing them to the respondents.

### Data Analysis

Data analysis was performed using SPSS (version 20; IBM Corporation) software. Frequency tables were prepared to assist in data cleaning. Data were presented using tables and charts with comparisons of different categories. Bivariate analyses were conducted to determine the relationships among the selected variables. Differences among categories were measured using the chi-square test. *P*<.05 were considered significant.

### Ethical Clearance

Ethical clearance was obtained from the institutional review board of MUHAS, Tanzania (reference number DA.282/298/01.C). Permission to implement this research was obtained from the Ministry of Health, Community Development, Gender, Elderly, and Children and the President Office Regional Administration and Local Government. Written informed consent was obtained from all participants. No identifiable personal information regarding the respondents was collected. The research data were kept secure and confidential by 2 researchers (DS and FS) and 2 data managers.

## Results

### General Characteristics

Responses from DHMs were received from all 186 district councils in the 26 regions in Tanzania Mainland. A total of 2667 (77.96%) of the expected 3421 DHMs responded, of which 2598 (97.41%) consented and completed the questionnaires. More than half (1466/2598, 56.43%) of the respondents were male, and approximately half (1291/2598, 49.69%) were aged between 35 and 48 years. Most participants (2038/2598, 78.44%) had a medical professional background (Table 1).

**Table 1:** Sociodemographics of participants (N=2598).

Characteristics			Values, n (%)
**Sex**			
	Female	1132 (43.57)	
	Male	1466 (56.43)	
**Age (years)**			
	20-34	852 (32.79)	
	35-49	1291 (49.69)	
	≥50	455 (17.51)	
**Profession**			
	Medical	2038 (78.44)	
	Nonmedical	560 (21.56)	
**Work experience (years)**			
	1-5	740 (28.48)	
	6-10	714 (27.48)	
	≥11	1144 (44.03)	
**Title**			
	Core district health management team members	1097 (42.22)	
	Program coordinators	834 (32.1)	
	Others	667 (25.67)	
**Education**			
	Degree holders	1312 (50.5)	
	Nondegree holders	1286 (49.5)	
**English proficiency**			
	Very fluent	572 (22.02)	
	Fluent	1221 (47)	
	Average	805 (31.98)	

### Perceived Usefulness of the DHIS2

DHMs were asked for their opinions on the usefulness of DHIS2. Most responses indicated that DHIS2 contributed to improved data quality (2239/2598, 86.18%) and reduced workload (2123/2598, 81.72%). Furthermore, 86.64% (2251/2598) of the respondents indicated that DHIS2 use has improved data availability (Multimedia Appendix 1).

### Opinions of Respondents on the Use of the DHIS2

As indicated in Table 2, DHMs were asked about their opinions on several issues pertaining to the usefulness of DHIS2 and the ease of use of the system. Overall, most DHMs expressed satisfaction with the system, explaining that the system was good for their work (2288/2598, 88.07%) and enabled them to be more effective (2187/2598, 84.18%). When asked if the system was confusing and difficult to learn and understand, most DHMs disagreed, as 66.05% (1716/2598) said it was not confusing and 75.17% (1953/2598) said it was not difficult to learn and understand.

**Table 2:** Opinions of respondents on the use of District Health Information Software (version 2; DHIS2; N=2598).

Opinion statements based on perception	Not sure, n (%)	Disagree, n (%)	Agree, n (%)
The DHIS2 enhances data use	233 (8.97)	126 (4.85)	2239 (86.18)
The DHIS2 enables effective completion of work	283 (10.89)	128 (4.93)	2187 (84.18)
The DHIS2 is good for my work	225 (8.66)	85 (3.27)	2288 (88.07)
The DHIS2 is confusing	527 (20.28)	1716 (66.05)	355 (13.66)
The DHIS2 is difficult to learn and understand	410 (15.78)	1953 (75.17)	226 (8.7)
Generally, I am satisfied with the DHIS2	321 (12.36)	203 (7.81)	2074 (79.83)

### Levels of Basic Computer Skills

For the DHMs to be able to use the DHIS2 as a tool, they ought to have skills in basic computer operations and applications and use of the DHIS2 software. Respondents were assessed using self-rated questions to establish their skill levels regarding various ICT issues. As indicated in Multimedia Appendix 1, slightly more than half (1377/2598, 53%) of the DHMs reported having advanced skills in basic computer operations, file management (1439/2598, 55.39%), Microsoft Word (1404/2598, 54.04%), and internet use (1298/2598, 49.96%). Almost all (2390/2598, 91.99%) members of the DHMT reported having either average or advanced skills in DHIS2 use.

A bivariate analysis was performed on some of the factors likely to be associated with respondents’ skills in DHIS2 use. Table 3 provides the association between respondents’ ability to use the DHIS2 and their sex, profession, title, and level of English proficiency. Male respondents, medical professionals, core members of the DHMT, and those with very high levels of proficiency in English were more likely to have higher skills in the use of the DHIS2 compared with their counterparts. The differences were statistically significant.

**Table 3:** Bivariate analysis of factors associated with respondents’ self-rated skill levels in District Health Information Software (version 2) use (N=2598).

Characteristics		Level of respondents’ skills in using the District Health Information Software (version 2), n (%)					*P* value	
		None	Average	Advanced	Total			
**Sex**								.005
	Male	95 (7)	883 (65.12)	378 (27.88)	1356 (100)			
	Female	113 (9.98)	733 (64.75)	286 (25.27)	1132 (100)			
**Age (years)**								.23
	20-34	75 (8.8)	564 (66.2)	213 (25)	852 (100)			
	35-49	90 (6.97)	855 (66.23)	346 (26.72)	1291 (100)			
	≥50	43 (7.89)	307 (56.33)	195 (35.78)	545 (100)			
**Profession**								.01
	Nonmedical	60 (10.71)	374 (66.78)	126 (22.5)	560 (100)			
	Medical	148 (7.26)	1352 (66.34)	538 (26.4)	2038 (100)			
**Experience (years)**								.56
	1-5	65 (8.78)	501 (67.7)	174 (23.51)	740 (100)			
	6-10	52 (7.28)	473 (66.25)	189 (26.47)	714 (100)			
	≥11	91 (7.95)	752 (65.73)	301 (26.31)	1144 (100)			
**Title**								.004
	Core district health management team members	78 (7.11)	701 (63.9)	318 (28.99)	1097 (100)			
	Program coordinators	70 (8.39)	558 (66.91)	206 (24.7)	834 (100)			
	Others	60 (9)	457 (68.52)	140 (20.99)	667 (100)			
**Education level**								.25
	Nondegree	102 (7.93)	837 (65.09)	347 (26.98)	1286 (100)			
	Degree	106 (8.14)	880 (67.54)	317 (24.33)	1303 (100)			
**English proficiency**								.001
	Very high (80%-100%)	52 (6.52)	446 (55.96)	299 (37.52)	797 (100)			
	High (60%-69%)	87 (7.07)	879 (71.46)	264 (21.46)	1230 (100)			
	Average (<59%)	69 (12.08)	401 (70.23)	101 (17.69)	571 (100)			

### Access to the DHIS2 and Training in Data Analyses

For DHMs to use DHIS2 data, they must have access to the DHIS2 and training on how to use it. Approximately half (1380/2598, 53.12%) of them reported having a user account and password to access the DHIS2. Slightly less than half (1237/2598, 47.61%) of the DHMs received training on data analysis using the DHIS2.

A bivariate analysis was performed on factors likely to be associated with receiving training in DHIS2 data analysis. Table 4 provides the association between training in DHIS2 data analysis and respondents’ sex, age, profession, experience at work, title, level of education, and level of proficiency in English. More male respondents, medical professionals, respondents with long experience at work, respondents who are program coordinators, nondegree holders, and respondents with a very high level of proficiency in English received training in DHIS2 data analysis.

**Table 4:** Bivariate analysis of factors associated with training in District Health Information Software (version 2) data analysis (N=2598).

Characteristics		Not trained, n (%)	Trained, n (%)	Total, n (%)	*P* value	
**Sex**						.004
	Male	732 (49.93)	734 (50.07)	1466 (100)		
	Female	629 (55.57)	503 (44.43)	1132 (100)		
**Age (years)**						.001
	20-34	499 (58.57)	353 (41.43)	852 (100)		
	35-49	650 (50.35)	641 (49.65)	1291 (100)		
	≥50	212 (46.59)	243 (53.41)	455 (100)		
**Profession**						.001
	Nonmedical	342 (61.07)	218 (38.93)	560 (100)		
	Medical	1019 (50)	1019 (50)	2038 (100)		
**Experience (years)**						.001
	1-5	444 (60)	296 (40)	740 (100)		
	6-10	381 (53.36)	333 (46.64)	714 (100)		
	≥11	536 (46.85)	608 (53.15)	1144 (100)		
**Title**						.001
	Core district health management team members	572 (52.14)	525 (47.86)	1097 (100)		
	Program coordinators	400 (47.96)	434 (52.04)	834 (100)		
	Others	389 (58.32)	278 (41.68)	667 (100)		
**Education level**						.001
	Nondegree	618 (48.06)	668 (51.94)	1286 (100)		
	Degree	743 (56.63)	569 (43.37)	1312 (100)		
**English proficiency**						.003
	Very high (80%-100%)	377 (47.3)	420 (52.7)	797 (100)		
	High (60%-69%)	673 (54.72)	557 (45.28)	1230 (100)		
	Average (<59%)	311 (54.47)	260 (45.53)	571 (100)		

### Ability of DHMs to Use DHIS2 Data

DHMs were asked to rate their skill levels based on their ability to enter data into the DHIS2 and use the data for decision-making. The ability to prepare league tables to rank the level of health facility performance was used as a proxy for data use. Approximately half (1287/2598, 49.54%) of the DHMs reported having an advanced level of skill for data entry, and approximately half (1321/2598, 50.85%) reported having average or advanced skills to prepare and use league tables. The proportion of team members with no skills to enter data or prepare and use league tables was very small (34/2598, 1.31% and 59/2598, 2.27%, respectively).

### Ability of DHMs to Use DHIS2 Modules and Data

DHMs were asked about their confidence in using the DHIS2 system and its data. Approximately two-thirds of the respondents had an average or high level of confidence in conducting data validation (1774/2598, 68.28%) and analyzing DHIS2 data to produce visualizations (1563/2598, 60.16%). Slightly less than three-quarters (1865/2598, 71.79%) of the team members had an average or high level of confidence in using DHIS2 data for planning. Of these team members, less than half were highly confident in conducting data validation (1151/2598, 44.3%) and using DHIS2 data for planning (1237/2598, 47.61%). Only one-third (937/2598, 36.07%) of them were highly confident in analyzing DHIS2 data and producing visualizations. Other areas in which data from DHIS2 are used included staff deployment and allocation of funds, medicine, and supplies.

Table 5 shows that the ability to use DHIS2 data (which was measured using ability to produce league tables as a proxy) was also associated with the respondent’s sex, age, profession, title, and level of proficiency in English. Male respondents, medical professionals, and those with a very high level of proficiency in English were more confident in using DHIS2 data compared with their counterparts, and the difference was statistically significant. However, unlike for DHIS2 use, most (1075/2598, 41.38%) of the core members of DHMTs who have skills in using both the DHIS2 software and data were program coordinators.

**Table 5:** Factors associated with the ability of district health managers to use District Health Information Software (version 2) data (N=2598).

Characteristics		Ability to use District Health Information Software (version 2) data, n (%)^a^						*P* value	
		None	Basic	Average	Advanced	Total			
**Sex**									.001
	Male	29 (1.98)	635 (43.32)	337 (22.99)	465 (31.72)	1466 (100)			
	Female	30 (2.65)	583 (51.5)	238 (21.02)	281 (24.82)	1132 (100)			
**Age (years)**									.11
	20-34	23 (2.7)	406 (47.65)	175 (20.54)	248 (29.12)	852 (100)			
	35-49	30 (2.32)	577 (44.69)	301 (23.32)	383 (29.67)	1291 (100)			
	≥50	6 (1.32)	235 (51.65)	99 (21.76)	115 (25.27)	455 (100)			
**Profession**									.01
	Nonmedical	10 (1.79)	296 (52.86)	117 (20.89)	137 (24.46)	560 (100)			
	Medical	49 (2.4)	922 (45.24)	458 (22.47)	609 (29.88)	2038 (100)			
**Experience (years)**									.049
	1-5	18 (2.43)	368 (49.73)	153 (20.68)	201 (27.16)	740 (100)			
	6-10	22 (3.08)	302 (42.3)	165 (23.11)	225 (31.51)	714 (100)			
	≥11	19 (1.66)	548 (47.9)	257 (22.47)	320 (27.97)	1144 (100)			
**Title**									.001
	Core district health management team members	22 (2.01)	472 (43.03)	238 (21.7)	365 (33.27)	1097 (100)			
	Program coordinators	19 (2.28)	394 (47.24)	185 (22.18)	236 (28.3)	834 (100)			
	Others	18 (2.7)	352 (52.77)	152 (22.79)	145 (21.74)	667 (100)			
**Education level**									.46
	Nondegree	32 (2.49)	592 (46.03)	299 (23.25)	363 (28.23)	1286 (100)			
	Degree	27 (2.06)	626 (47.71)	276 (21.04)	383 (29.19)	1312 (100)			
**English proficiency**									.001
	Very high (80%-100%)	18 (2.26)	283 (35.51)	165 (20.7)	331 (41.53)	797 (100)			
	High (60%-69%)	26 (2.11)	598 (48.62)	292 (23.74)	314 (25.53)	1230 (100)			
	Average (<59%)	15 (2.63)	337 (59.02)	118 (20.67)	101 (17.67)	571 (100)			

^a^The ability to produce league tables was used as a proxy to measure the ability to use District Health Information Software (version 2) data.

### Existence of a Supporting Environment for the Use of the DHIS2

In general, approximately 59.85% (1555/2598) of the respondents indicated the need for more resources to support the effective use of DHIS2. More than half of the respondents did not agree that their councils had an adequate number of computers (1504/2598, 57.89%) and reliable internet connection to support DHIS2 functions (1485/2598, 57.16%). Slightly less than half (1227/2598, 47.23%) of the team members disagreed that their councils had an adequate budget to run the DHIS2.

### Perceived Challenges Hindering the Implementation and Use of the DHIS2

Respondents were asked to indicate the challenges affecting the implementation and effective use of the DHIS2. Table 6 shows that most of the respondents (1890/2598, 72.75%) felt that overwhelming paperwork was the main constraint affecting the implementation of the DHIS2. Approximately two-thirds (1670/2598, 64.28%) of the respondents felt there was inadequate connectivity and ICT support (1734/2598, 66.74%), approximately 59.74% (1552/2598) felt that slow internet speed was a serious problem, and approximately half (1307/2598, 50.31%) thought data quality was a challenge.

**Table 6:** Perceived challenges hindering the implementation and use of the District Health Information Software (version 2; N=2598).

Challenges affecting the use of District Health Information Software (version 2)	Participants, n (%)
There is a lot of paperwork	1890 (72.75)
Inadequate technical support	1566 (60.28)
Inadequate information and communication technology officers	1734 (66.74)
Unreliable internet connectivity	1670 (64.28)
Slow internet speed	1552 (59.74)
Data quality compromised during data processing	1307 (50.31)
Lack of guidelines for filling out the main data sources and reporting forms	1190 (45.8)
Data collection and reporting forms are not standardized; some groups have their own formats	1028 (39.57)
Lack of feedback	986 (37.95)
Electrical power interruption or unreliable electricity	895 (34.45)
Parallel data systems collecting the same indicators	814 (31.33)
Data collection and reporting forms are not standardized; some groups have their own formats	733 (28.21)
Data overload: data management operational processes are not documented	675 (25.98)
Personnel are not trained in the use of data sources and reporting forms	596 (22.94)

## Discussion

### Principal Findings

In this study, most (2074/2598, 79.83%) DHMs reported satisfaction with the DHIS2, stating that it improved their quality of work and made their daily work effective. This study shows that DHMs have appreciable competencies in using the DHIS2 and its data. Some challenges have been identified, and recommendations have been made for the improvement of access to and use of DHIS2 data. Similar findings have been reported in other countries that implemented the DHIS2, where health workers were reported to be satisfied with the ease of data processing [11,23,24]. In addition, the perception of health workers regarding the use of health information technology, such as DHIS2, is influenced by user-friendliness and the perceived benefits of the system [25]. Although in these studies the reported satisfaction was limited to the use of the application (in data processing), our study went further to reveal satisfaction with the outcomes of using the application. Furthermore, in this study, >80% of the DHMs in Tanzania indicated a high level of commitment to using the DHIS2, as they perceived the system to have improved data quality, reduced workload, and improved data use and decision-making (Table 2). This is above the commitment levels reported in other studies in Ethiopia, Ghana, Nigeria, and Iran [26].

The high rate of satisfaction reported in our study was attributed to the improved availability and quality of routine data and the reduction in workload burden for DHMs. Before the introduction of the DHIS2, availability and quality of routine data were unsatisfactory [13,27]. Reporting systems were weak, and user satisfaction with usability was low. The situation changed after the introduction of the DHIS2, where completeness and timeliness of reporting were maintained at a very high rate [28]. Improved data availability can be attributed to the web-based feature of DHIS2 with a centralized server that simplifies data entry and access and enables anyone with access rights to view the data from anywhere [8,11]. In Tanzania, the DHIS2 has integrated >15 intervention programs and several digital HISs, including human resources for HISs and health facility registries [16]. The creation of a central data warehouse on a central web-based server integrated with other digital HISs makes the DHIS2 a robust source of information and ensures better access to data and information by users [11,24,29-31].

The DHMs’ satisfaction with data quality emanates from the DHIS2 data validation tools that simplify consistency checks compared with when the exercise was performed on a paper-based system [9]. As the DHIS2 brings together data from various programs, data triangulation can be performed easily and discrepancies, identified [15]. Moreover, the ease in accessing DHIS2 data has exposed more people to it, which has facilitated feedback and self-assessment, making it easier for DHMs to identify errors for prompt correction [4,24].

DHMs’ satisfaction with reduced workload burden can be explained by the ability of the DHIS2 modules to produce standardized tables and allow tailor-made reports to be prepared by users using pivot tables [9].

The high level of satisfaction with the software reflects the readiness of DHMs to develop further, which can be taken as an opportunity for the DHIS2 implementers or Ministry of Health to strengthen the strategy for increasing data demand and use, which is currently a major challenge [32].

Almost all (2390/2598, 91.99%) DHMs reported having at least basic-level skills on the DHIS2, whereas half of them (664/2598, 25.56%) reported having advanced skills. To our knowledge, this is the first study to quantify and report the competencies of health managers in using the DHIS2 and data generated from it.

One of the major factors that might have contributed to such a level of competency is the introduction of the DHIS2 on an existing paper-based HISs, which was relatively well developed with functioning structures and materials for data collection, reporting, and analysis [33]. In addition, the training of DHMs was based on hands-on practice for data analysis using actual data, the provision of technical support and supportive supervision, and the distribution of computers and accessories to all districts immediately after the training. Furthermore, the high level of competencies is likely to be because of the decentralization of governance in the districts, which has provided them authority for decision-making in the planning and implementation of interventions [34]. The extensive use of data from the DHIS2 during the preparation of Comprehensive Council Health Plans through the Planning and Reporting system (PlanRep) [35] may also explain why more DHMs reported a higher level of competency in the use of DHIS2 data for planning compared with other purposes.

Program coordinators received more training in data analysis compared with other members of the DHMT. This can be explained by the fact that training for continuing education in many districts is supported by implementing and development partners through their respective intervention programs. In such training, program managers are given priority, whereas other members are often left out because of limited funding.

In this study, only half (1380/2598, 53.12%) of the DHMs reported having access rights to the DHIS2 application. Similar findings were reported in a study conducted in Kenya, where approximately half of the health workers in the studied health facilities reported having no access rights, and as a result, they did not use the DHIS2 data [4]. Challenges in having access rights undermine the strength of the DHIS2 in ensuring that DHMs have prompt access to information for decision-making [8,11]. Nevertheless, we report a higher proportion of DHMs using the DHIS2 than those reporting to have DHIS2 user accounts. In addition to sharing accounts among DHIS2 users, this may also indicate peer or self-learning, a phenomenon that is uncommon in the country’s health sector [18].

Finally, we report challenges in the provision of a supporting environment that is likely to have undermined the competency of DHMs in using the DHIS2 application and the generated data. These include inadequate support for ICT, including computers and internet connections and insufficient funds at the district level. Interestingly, the level of achievement attained with only half of the expected resources is appreciably high. This indicates that there is a high potential for improvement if more resources are available to DHMs.

### Implications of the Study Findings

Training in DHIS2 use has been cited in the literature as one of the challenges affecting the establishment and effective use of the system [10]. The fact that almost all (2390/2598, 91.99%) DHMs reported having at least basic computer and DHIS2 application skills provides a favorable environment for upgrading to the advanced level. As the proportion of DHMs who reported having advanced skills in the use of DHIS2 data was dismal, it is still adequate to form a critical mass that can be used to prepare data use champions. These can then be deployed to provide on-the-job peer education; supportive supervision; and mentoring to all members of the DHMT, irrespective of their cadre; with the aim of strengthening the organizational culture of data demand and use for decision-making [36].

DHMs play a central role in the planning and implementation of primary health care services in a decentralized country such as Tanzania [34]. The reported competency in using DHIS2 data found in this study is likely to have a multiplier effect at the health facility level, where decisions made by DHMs have a profound effect, and at the national level, where DHMs are held accountable for resources invested in district health services.

### Methodological Considerations of This Study

The findings of this study are self-reported; hence, they are likely to be overestimated for respondents who want to exaggerate their capacity in the hope of being applauded or may underestimate their capacity in the hope of receiving more support. Studies have indicated that respondents prefer to attend training or meetings because of the allowances paid—the *per diem syndrome* [37-39]. In this case, some respondents may prefer to underestimate their level of knowledge so that they can get opportunities to attend training for the sake of receiving allowances. Therefore, it is difficult to predict the direction of bias likely to result from the reported data.

The web-based administration of questionnaires enables access to large and different populations, and it is an inexpensive, quick, and convenient approach to collecting data [40-42]. However, this approach has the disadvantage of having relatively lower response rates compared with face-to-face administration [40]. Nevertheless, in this study, the response rate was higher (75.9%) than the mean response rate for web-based data collection approaches reported by Blumenberg and Barros [40].

Furthermore, the scope of this study was limited to determining DHMs’ perceived competency in using DHIS2 and the generated data. The quantitative nature of this study prevented us from answering some pertinent practical questions. Questions such as “What is behind the reported success in Tanzania?” will require qualitative studies to answer them.

### Strengths of Our Study

This is the first study to report the competencies of DHMs in using the DHIS2 application and the generated data. This study gathered primary data from the DHMs in 186 districts in the country. In this study, we quantified the level of satisfaction with the DHIS2 application among DHMs, its usefulness, and their competencies in using the application and generated data.

### Conclusions

DHIS2 modules have enhanced availability and quality of data and reduced workload burden compared with the situation that existed before its commencement. This has led to satisfaction with the application by most (2187/2598, 84.18%) DHMs because of improved quality of work and their effectiveness. Although almost all (2564/2598, 98.69%) DHMs reported to have at least basic skills on DHIS2, approximately one-fourth of them (746/2598, 28.71%) reported having high-level skills. The Ministry of Health needs to seize this opportunity to train the few DHMs with high-level skills to enable them to support the majority who have basic-level skills in data use for decision-making. Further research is needed to determine the factors that influence the reported DHMs’ competency and the extent to which the competencies are translated into data use for decision-making to improve health care services.

## Appendix

Multimedia Appendix 1 Supplementary Tables 1-6.

## Abbreviations

DHIS2: District Health Information Software (version 2)
DHM: district health manager
DHMT: district health management team
HIS: health information system
HMIS: health management information system
ICT: information and communication technology
MUHAS: Muhimbili University of Health and Allied Sciences
