# Supplementary material for: Perceived Usefulness, Competency, and Associated Factors in Using District Health Information System Data Among District Health Managers in Tanzania: Cross-sectional Study
Source: JMIR Form Res. 2022 May 23;6(5):e29469. doi: 10.2196/29469 (PMC9171597; doi:10.2196/29469)
Supplement: Multimedia Appendix 1 [file formative_v6i5e29469_app1.docx]

**Multimedia Appendix 1: Supplementary File**

**Table S1: Perceived usefulness of the DHIS2 (n=2598)**

| **Statement, n (%)** | **Not sure** | **Disagree** | **Agree** |
| --- | --- | --- | --- |
| DHIS2 use has improved data availability | 227 (8.74) | 120 (4.62) | 2251 (86.64) |
| DHIS2 use has improved data quality | 233 (8.97) | 126 (4.85) | 2239 (86.18) |
| DHIS2 use has reduced workload | 311 (11.97) | 164 (6.31) | 2123 (81.72) |

**Table S2: Self-rated level of ICT skills among District Health Managers (n=2598)**

| **Item** | **None, n (%)** | **Average, n (%)** | **Advanced, n (%)** |
| --- | --- | --- | --- |
| Basic computer operations e.g., keyboard, mouse | 17 (0.65) | 1204 (46.34) | 1377 (53.00) |
| File management (create, open, save, etc.) | 23 (0.88) | 1136 (43.73) | 1439 (55.39) |
| Word processing (e.g., Microsoft Word) | 21 (0.81) | 1173 (45.15) | 1404 (54.04) |
| Spreadsheets (e.g., Microsoft Excel) | 51 (1.96) | 1712 (65.90) | 835 (32.14) |
| PowerPoint presentations (e.g., Microsoft PowerPoint) | 39 (1.50) | 1457 (56.08) | 1102 (42.42) |
| Internet surfing and searching | 29 (1.12) | 1271 (48.92) | 1298 (49.96) |
| Email communication | 16 (0.62) | 1089 (41.92) | 1493 (57.46) |
| DHIS2 usage | 208 (8.01) | 1726 (66.44) | 664 (25.56) |

**Table S3: Access to the DHIS2 and training in data analysis (n=2598)**

| **Characteristic** | | **Participants, n (%)** |
| --- | --- | --- |
| Do you have a user account to access the DHIS2?**,** | No | 1218 (46.88) |
|  | Yes | 1380 (53.12) |
| Have you received training on DHIS2 data analysis? | No | 1361 (52.39) |
|  | Yes | 1237 (47.61) |

**Table S4: Ability to enter data in the DHIS2 and use it to prepare league tables (n=2598)**

| **Characteristic, n (%)** | **None** | **Basic** | **Average** | **Advanced** |
| --- | --- | --- | --- | --- |
| Ability to enter data in the DHIS2 | 34(1.31) | 719(27.68) | 558(21.48) | 1287(49.54) |
| Ability to use data and prepare league tables | 59(2.27) | 1218(46.88) | 575(22.13) | 746(28.71) |

**Table S5: Perceived adequacy of supporting systems / environment (n=2598)**

| **Opinion statements, n (%)** | **Not sure** | **Disagree** | **Agree** |
| --- | --- | --- | --- |
| My District council has adequate computers to enable the DHIS2 | 456 (17.55) | 1504 (57.89) | 638 (24.56) |
| My District council has adequate ICT personnel to support the use of the DHIS2 | 530 (20.40) | 1555 (59.85) | 513 (19.75) |
| My District council has an adequate budget for the DHIS2 | 789 (30.37) | 1227 (47.23) | 582 (22.40) |
| My District council has reliable internet connection to support DHIS2 functions | 477 (18.36) | 1485 (57.16) | 636 (24.48) |

**Table S6: District Health Managers’ ability to use DHIS2 modules and data (n=2598*)**

|  | **Level of confidence, n (%)** | | |
| --- | --- | --- | --- |
| **Statement** | **Low** | **Average** | **High** |
| I am capable of navigating through the DHIS2 | 704 (27.37) | 609 (23.68) | 1259 (48.95) |
| I am capable of entering data into the DHIS2 | 719 (28.04) | 558 (21.76) | 1287 (50.20) |
| I am capable of doing data validation in the DHIS2 | 787 (30.73) | 623 (24.33) | 1151 (44.94) |
| I am capable of analysing data in the DHIS2 and producing visualizations | 1004 (39.11) | 626 (24.39) | 937 (36.50) |
| I am capable of using DHIS2 data for staff deployment | 951 (37.12) | 622 (24.28) | 989 (38.60) |
| I am capable of using DHIS2 data for allocation of funds | 966 (37.66) | 625 (24.37) | 974 (37.97) |
| I am capable of using DHIS2 data for procurement of medicine and supplies | 1030 (40.28) | 573 (22.41) | 954 (37.31) |
| I am capable of using DHIS2 data for reporting | 655 (25.58) | 583 (22.76) | 1323 (51.66) |
| I am capable of using DHIS2 data for planning | 695 (27.15) | 628 (24.53) | 1237 (48.32) |
| I am capable of using DHIS2 data to provide feedback | 660 (25.89) | 605 (23.73) | 1284 (50.37) |
| I am capable of using DHIS2 data for policy decisions | 973 (38.19) | 630 (24.73) | 945 (37.09) |

*Denominator/number of respondents vary for different statements
